# Importance of a detailed anomaly scan after a cfDNA test indicating fetal trisomy 21, 18 or 13

**DOI:** 10.1007/s00404-023-07311-2

**Published:** 2023-12-13

**Authors:** Tobias Spingler, Jiri Sonek, Markus Hoopmann, Natalia Prodan, Gertruda Jonaityte, Tania Elger, Karl Oliver Kagan

**Affiliations:** 1 Department of Women’s Health, University of Tuebingen, Calwerstrasse 7, 72076 Tübingen, Germany; 2 Fetal Medicine Foundation USA, Dayton, OH USA; 3 Division of Maternal Fetal Medicine, Wright State University, Dayton, OH USA

**Keywords:** Fetal defects, cfDNA, NIPT, Trisomy, Ultrasound examination

## Abstract

**Objective:**

To investigate the effect of the presence or absence of fetal anomalies and soft markers diagnosed by ultrasound on positive predictive value (PPV) 21, 18 and 13 in pregnancies with a high-risk cfDNA result.

**Methods:**

Retrospective study including singleton pregnancies with high-risk NIPT results for common trisomies followed by invasive testing. The cases were grouped by gestational age at the time of invasive testing and by the presence or absence of fetal abnormalities or soft markers. The ultrasound was considered abnormal if at least one major defect or a soft marker was detected.

**Results:**

A total of 173 women were included. Median maternal and gestational age was 37.7 years and 14.0 weeks, respectively. CfDNA test result showed high-risk for trisomy 21 and trisomy 18 or 13 in 119 and 54 cases, respectively. The “pre-ultrasound” PPV for trisomy 21 and for trisomy 18 or 13 were 98.3% and 68.4%, respectively. In case of a high-risk result for trisomy 21 and no fetal anomalies, the PPV was 86.7% while it was 100% if there were anomalies or markers present. In the case of a high-risk result for trisomy 18 or 13, the PPV was 9.5% if the ultrasound examination was normal and 100% if the ultrasound examination was abnormal.

**Conclusion:**

This study suggests that a detailed ultrasound examination performed after a cfDNA result that is high-risk for one of the common autosomal trisomies adds significantly to establishing an individualized risk assessment. This is particularly true in cases with a high-risk result for trisomies 18 or 13.

## What does this study add to the clinical work?

**Table:** 

CfDNA screening for trisomy 21, 18 and 13 has an excellent detection- and false positive rate. However, the PPV is lower and dependent on multiple other parameters, such as the prevalence of a certain disease. In this study, we show that the ultrasound examination after the cfDNA test is important to estimate the PPV.	

## Introduction

Cell-free DNA testing (a.k.a. cfDNA screening or NIPT) has revolutionized prenatal screening for the common autosomal trisomies [1]. A recent meta-analysis by Rose et al. found that in the general population, the detection rate for both trisomy 21 and 18 is 98.8% and for trisomy 13 it is 100% [2]. For all three trisomies, the authors reported false-positive rates of 0.04–0.07%. In counseling patients with a high-risk cfDNA result, the positive predictive value (PPV) plays an important role. In the same meta-analysis, the PPV for trisomy 21 was 91.8%. It was 65.8% and 37.2% for trisomy 18 and 13, respectively [2].

In addition to the patients’ age and history, the PPV is also dependent on the presence or absence of fetal defects on ultrasound. Wagner et al. showed that 83% of trisomy 18 fetuses and all trisomy 13 fetuses had at least one major defect that was identified in the first trimester [3]. In the second trimester, 89% and 83% of fetuses with trisomy 18 and 13, respectively, had at least one anomaly [4, 5]. Because many fetuses with trisomy 21 do not have major defects, identification of affected fetuses often relies on the assessment of first and second trimester soft markers [6]. Abele et al. showed that in the first trimester 96% of fetuses with trisomy 21 had an increased risk based on nuchal translucency thickness and additional markers [7]. In the second trimester, Agathokleous et al. reported that about 69% of the affected fetuses had at least one soft marker [6]. On the other hand, in the absence of fetal defects, it is very unlikely that a fetus has trisomy 18 or 13. In the case of trisomy 21, the absence of defects does not exclude this condition, but the likelihood is reduced.

In this study, we aimed to investigate the effect of the presence or absence of fetal anomalies and soft markers diagnosed by ultrasound on the PPV in pregnancies with a high-risk cfDNA result for trisomies 21, 18, and 13.

## Methods

This is a retrospective study consisting of pregnant women with a high-risk NIPT result for trisomy 21, 18 and 13 who were seen at the Department of Prenatal Medicine, University Hospital Tuebingen, Germany between 2016 and 2023. Women who were involved in this study were either referred for a risk assessment for common autosomal trisomies or due to a high-risk cfDNA test result. The data of some of the women in the current study were included in our previous studies dealing with other aspects of cfDNA screening in pregnancy [8–13]. The cfDNA test used in most of the cases was Harmony^®^ Prenatal Test (Roche Inc, San Jose, California).

Our standard management protocol of women with a high-risk NIPT result includes genetic counselling and a detailed ultrasound examination performed according to national and international guidelines [14–17]. An invasive test is always offered. If no fetal defects or markers are detected, we offer an amniocentesis. If markers or fetal anomalies are seen in the first trimester, chorionic villus sampling (CVS) is offered [18–21]. If a woman decides to continue the pregnancy regardless of karyotype, we inform her that postnatal karyotyping is available. In all cases, the management is based on the woman's personal decision. The karyotype is determined by a cytogenetic analysis. This study included only women with singleton pregnancies who had undergone the standard treatment protocol.

In this study, we grouped cases by gestational age at the time of invasive testing (< 14 + 0 weeks’ gestation versus ≥ 14 + 0 weeks’ gestation) and by the presence or absence of fetal abnormalities or markers for autosomal trisomies at the time of invasive testing. The ultrasound examination prior to the invasive test was considered abnormal if at least one major defect or a soft marker was detected.

The following findings were considered as second trimester soft markers: intracardiac echogenic focus, mild ventriculomegaly, increased nuchal fold, echogenic bowel, mild hydronephrosis, short humerus and/or femur, aberrant right subclavian artery, absent or hypoplastic nasal bone [6]. In addition, fetal growth restriction, a flat face and retrognathia were considered as soft markers. Fetal defects were classified as anomalies if they are known to require postnatal therapy. In the first trimester, a nuchal translucency thickness of ≥ 3.5 mm (99th percentile) was classified as an anomaly. A nuchal translucency thickness between the 95th percentile and 3.5 mm, an absent nasal bone, and an abnormal blood flow in either the ductus venosus or across the tricuspid valve were considered to be markers.

The results of the ultrasound examination and the corresponding images are stored in the Viewpoint database (Viewpoint General Electrics, Munich/Germany).

This study was approved by the ethical committee at the University Hospital Tuebingen, Germany (695/2023BO2).

### Statistical analysis

Maternal characteristics are shown by median and interquartile range. The positive predictive value was calculated by dividing the number of screen positive cases with an abnormal karyotype by the number of all screen positive cases. Differences were compared by chi square test, Student’s *t*-test or by Mann–Whitney *U* test. 95% Confidence intervals were calculated based on the Clopper-Pearson method. Uni- and multivariable logistic regression analyses were used to identify covariates that are significantly associated with a true positive cfDNA high-risk result. We first examined the impact of maternal and gestational age, maternal weight, and of the fetal fraction by a univariable logistic regression. In a second step, the parameters that were found to be significantly associated with a true positive cfDNA high-risk result were further assessed by a multivariable logistic regression analysis (stepwise backwards: Wald). The *p* level of 0.05 or less was considered as significant.

## Results

The study population consisted of 173 women with a high-risk cfDNA result for trisomies 21, 18, or 13. In 119 cases, the cfDNA test result was high-risk for trisomy 21 and in 54 the risk was increased for trisomy 18 or 13. Median maternal and gestational age was 37.7 years and 14.0 weeks, respectively. In 86 (49.7%) women, the ultrasound examination before invasive testing was carried out prior to 14.0 weeks and in 87 (50.3%) women beyond that point. CVS was done in 112 (64.7%) pregnancies and an amniocentesis in 61 (35.3%) cases.

A detailed description of the study population is shown in Table 1. The median fetal fraction was 9.7%. Women with a false positive result for trisomy 18 and 13 were significantly younger than those with a true positive result and the fetal fraction was lower. In the group of fetuses with a false and true cfDNA test indicating trisomy 21, there was also a significant difference in the fetal fraction with the euploid pregnancies having a lower fetal fraction.

**Table 1:** Detailed description of the study population

	CfDNA test indicating Trisomy 18/13		CfDNA test indicating Trisomy 21	
	Euploid	Abnormal karyotype	Euploid	Abnormal karyotype
*n*	19	35	2	117
Maternal age median (IQR)	34.3* (33.2–37.6)	39.1* (35.1–41.3)	38.1 (34.3–41.9)	37.7 (34.4–40.5)
Gestational age median (IQR)	15.5 (14.0–16.0)	13.6 (12.4–15.6)	15.2 (15.0–15.4)	13.9 (12.7–14.9)
Maternal weight median (IQR)	65.0 (60.4–76.9)	66.5 (60.0–75.0)	72.6 (72.5–72.6)	64.5 (59.0–75.0)
Fetal fraction median (IQR)	5.7** (4.6–6.9)	7.6** (6.5–10.0)	5.7*** (4.6–6.9)	10.9*** (8.2–13.6)

*Student’s *t*-test *p* = 0.031, **Student’s *t*-test *p* = < 0.001, ***Man–Whitney-*U*-test *p* = 0.009

The karyotype was abnormal in 117 of the 119 pregnancies with a high-risk cfDNA result for trisomy 21. Thus, the overall observed PPV for trisomy 21 prior to the ultrasound examination was 98.3% (95% CI 94.1–99.8%).

**Fig. 1:**
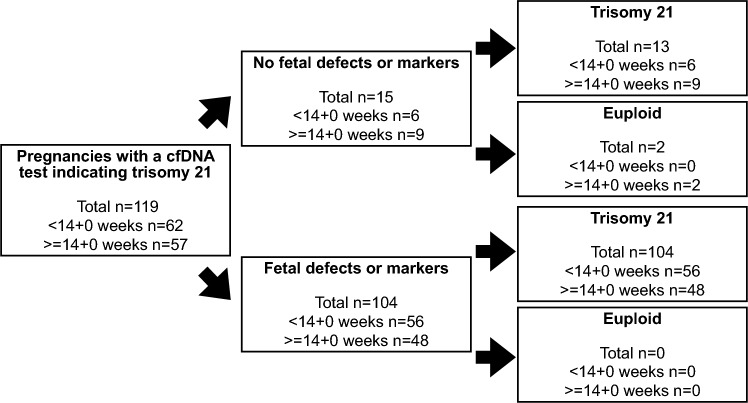
Distribution of fetuses with and without trisomy 21 stratified according to the presence or absence of fetal anomalies

In 15 cases, the fetal anatomy was normal without anomalies or markers. Of those, diagnostic testing showed trisomy 21 in 13 fetuses and two were euploid (PPV for trisomy 21: 86.7%, 95% CI 59.5–98.3%). In 104 cases, the ultrasound examination found either anomalies or markers and all these fetuses had trisomy 21 (PPV 100%, 95% 96.5–100%) (Fig. 1 and Table 2). The PPV for trisomy 21 in cases with no anomalies or markers was significantly lower than the PPV in cases with anomalies or markers (Chi square test *p* < 0.001). The abnormal findings are listed in Table 3.

**Table 2:** Positive predictive values for trisomy 21, 18 or 13 in cases with high-risk cfDNA result for trisomy 21 and trisomy 18 or 13 stratified according to the result of the ultrasound examination

Positive predictive value for trisomy	High risk cfDNA result	
	For trisomy 21 (*n* = 119)	For trisomy 18 or 13 (*n* = 54)
Prior to the ultrasound examination	98.3% (*n* = 119)	68.4% (*n* = 54)
After the ultrasound examination		
Present anomalies or markers	100% (*n* = 104)	100% (*n* = 33)
No anomalies or markers	86.7% (*n* = 15)	9.5% (*n* = 21)

**Table 3:** Fetal anomalies and markers in fetuses with trisomy 21

Fetal anomalies and markers in fetuses with trisomy 21 (*n* = 117)	*n*	%
Anomalies		
NT ≥ 3.5 mm	37	31.6
Heart	34	29.1
Kidneys	5	4.3
Extremities	3	2.6
Abdominal wall	2	1.7
CNS	1	0.9
Markers		
Nasal bone	52	44.4
Ductus venosus	38	32.5
Tricuspid blood flow	23	19.7
NT between 95th centile and 3.5 mm	18	15.4
Echogenic intracardiac focus	11	9.4
Fetal growth restriction	1	0.9
Single umbilical artery	3	2.6
Abnormal fetal profile	8	6.8
Aberrant right subclavian artery	3	2.6

A fetus can more than *one* anomaly or marker. The percentages refer to the overall number of affected fetuses

**Fig. 2:**
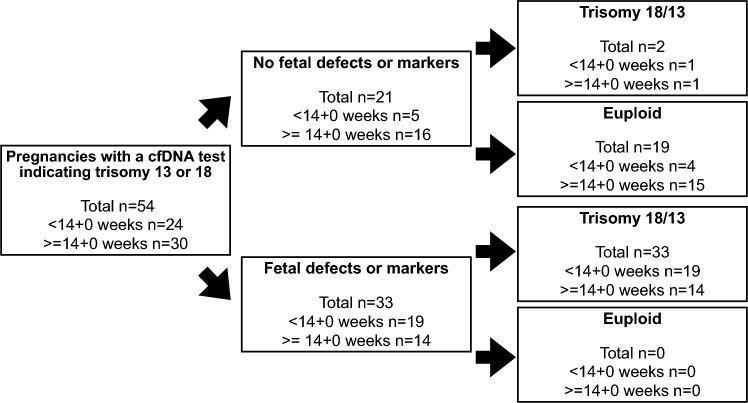
Distribution of fetuses with and without trisomy 18/13 stratified according to the presence or absence of fetal anomalies

In 54 pregnancies, the cfDNA test showed an increased risk for trisomy 18 or 13. The karyotype was abnormal in 35 cases. Thus, the “pre-ultrasound” PPVfor these two trisomies was 68.4% (95% CI 50.6–77.3%). In 33 fetuses, there were anomalies or markers, and all fetuses were aneuploid (PPV for trisomy 18 or 13: 100%, 89.4–100%) (Tables 2 and 4; Fig. 2). There were 21 pregnancies with a normal ultrasound examination. In this group, 2 fetuses had trisomy 18 (PPV for trisomy 18 or 13: 9.5%, 95% CI 1.2–30.4%) while 19 (90.5%) fetuses were euploid. The difference between the PPV for trisomy 18 or 13 in the two groups was highly significant (Chi square *p* < 0.001). Of note is that one of the two trisomic fetuses without fetal defects had a mosaic trisomy 18 while the other fetus had a full trisomy 18. In the first case, free beta-hCG and PAPP-A were 0.14 MoM and 0.17 MoM, respectively; therefore, the pregnancy was classified as high-risk based on the serum markers even without ultrasound signs. The serum marker levels in the other pregnancy were not measured.

**Table 4:** Fetal anomalies and markers in fetuses with trisomy 18 and 13

Fetal anomalies and markers in fetuses with trisomy 18 or 13 (*n* = 35)	*n*	%
Anomalies		
NT ≥ 3.5 mm	5	14.3
Facial clefts	5	14.3
Heart	23	65.7
Kidneys	4	11.4
Extremities	11	31.4
Abdominal wall	14	40.0
CNS	6	17.1
Markers		
Nasal bone	6	17.1
Ductus venosus	10	28.6
Tricuspid blood flow	4	11.4
NT between 95th centile and 3.5 mm	2	5.7
Fetal growth restriction	7	20.0
Single umbilical artery	8	22.9
Abnormal fetal profile	8	22.9

A fetus can more than *one* anomaly or marker. The percentages refer to the overall number of affected fetuses

We used uni- and multivariable regression analysis to determine significant covariates for a true positive cfDNA result (Table 5). We were unable to include the results of the ultrasound examination in the regression analysis as there were no fetuses with anomalies or markers in this study that were euploid; therefore, the only significant contributor for a true positive result was a high fetal fraction level.

**Table 5:** Uni- and multivariable regression analysis to identify covariates that are significantly associated with a true positive result

	Univariate		Multivariate	
	OR	*p*	OR	*p*
Maternal age in years	1.111 (1.001–1.233)	0.049	1.120 (0.982–1.128)	0.091
Gestational age in weeks	0.927 (0.829–1.037)	0.186		
Maternal weight in kg	0.987 (0.957–1.019)	0.827		
Fetal fraction in %	2.391 (1.597–3.581)	< 0.001	**2.436 (1.607–3.692)**	**0.008**

## Discussion

In this study, we have shown that a detailed ultrasound examination done after a cfDNA test result that indicates high-risk for the common autosomal trisomies can be used to adjust the PPV for trisomy. This is especially true for trisomy 18 or 13. In these cases, the PPV was only 11% when the ultrasound examination was normal and 100% when fetal abnormalities or markers were present. The PPV for trisomy 21 was also dependent on the presence or absence of fetal anomalies or makers, but to a lesser extent.

Our results are consistent with the few previous studies that are available. Scott et al. investigated the PPV of a first trimester ultrasound scan in 612 cases where a cfDNA test result indicated high risk of aneuploidy. [22]. Following a normal first trimester ultrasound examination, the PPV for trisomies 21, 18, 13 and monosomy X were 68%, 57%, 5% and 25%, respectively. In contrast, in cases where fetal defects were found, the PPV was close to 100%. In our study population, we found an even more pronounced reduction in the PPV for trisomy 18 and 13 after a normal ultrasound examination. Zhen et al. studied 81 cases with a cfDNA test indicating high-risk for trisomy 18 and 13 in the first and second trimesters [23]. The authors reported that the PPV of the cfDNA test prior to an ultrasound examination was 60.7% for trisomy 18 and 30% for trisomy 13. When the NIPT was supplemented by ultrasound, the new PPV for trisomy 18 was 100% if fetal defects were present. In contrast, the negative predictive value of a normal ultrasound examination was 92.3%. In the group of pregnancies where the cfDNA test indicated trisomy 13, the result of the ultrasound examination was even more helpful. All fetuses with abnormalities were aneuploid and all normal fetuses euploid. These results are similar to our findings.

Interestingly, in the current study the fetal fraction was found to be a significant contributor to a true or false positive result. Higher fetal fraction levels were more often associated with a true positive result. In a recent study, we examined the results of nearly 400,000 cfDNA tests and found that in screening for trisomy 21, the screen-positive rate was relatively constant, regardless of the fetal fraction [24]. In contrast, in screening for microdeletion 22q11.2, we found a strong association between the fetal fraction and the screen positive rate [25]. Wright et al. used a theoretical model and found a decreasing detection rate for trisomy 21 with lower fraction levels [26].

There are many arguments that can be made in favor of a detailed ultrasound examination before any cfDNA test and after an abnormal test result [14, 15, 27, 28]. First, the prevalence of fetal structural defects is much higher than the prevalence of chromosomal abnormalities, especially of those that can be detected by cfDNA screening [1]*.* The best method to detect fetal anomalies is a detailed ultrasound examination. Second, fetal defects are associated with a wide spectrum of chromosomal abnormalities. A cfDNA test can be useful when looking for a specific disease but it cannot rule out all chromosomal abnormalities that may be associated with fetal anomalies. Maja et al. examined the residual risk of a genetic disease being present after a normal cfDNA test and found that, even after a genome wide cfDNA screening test, there was still a 0.7% risk for a chromosomal abnormality [29]. Third, cfDNA testing has an excellent test performance in screening for trisomy 21, 18 and 13, but is also associated with a relatively high risk of test failure (up to 5%) [24, 30]. In cases of test failure, it is generally recommended to offer invasive testing because of the increased risk of chromosomal abnormalities that is associated with a low fetal fraction, especially for trisomy 18, 13 and triploidy. If the ultrasound examination in the setting of a failed cfDNA test is normal, the chance of normal fetal development is so high that it is reasonable to begin to question the need for invasive testing to exclude trisomy 18, 13 and triploidy. Fourth, a contingent screening approach, such as starting with a combined first trimester screen and an early fetal anatomic assessment that is followed by cfDNA testing done only under certain conditions may be utilized. If cfDNA testing is done in a smaller group of women such as those that are at an intermediate risk for trisomy 21, this approach might prove to be more cost-effective than universal cfDNA screening [28]. Under these conditions, one could even question if a cfDNA test for trisomy 18 and 13 isuseful if the anatomical assessment is normal.

Our study has some limitations. First, the median maternal age of the cohort studied was relatively advanced. This increases the “pre-ultrasound” PPV for trisomy due to the higher prevalence of the disease. The contribution of the ultrasound examination may even be greater in a younger population. Importantly, the study was carried out in a single tertiary center where women are referred not only for risk assessment for common trisomies but also for further management of cases with a high-risk cfDNA test result. This explains in part the fact that in our study the PPV was 98% in screening for trisomy 21 which is significantly higher than the PPV of 92% (95% CI 88.4–94.2%) found in the meta-analysis of Rose et al. [2]. Also, it is likely that in some of the cases referred to us, the local obstetrician initially found a fetal anomaly which then was followed by a cfDNA test, and a referral was made only if the result indicated high-risk for aneuploidy. However, this does not weaken the aim of this study which was to demonstrate the importance of a detailed ultrasound examination after a high-risk cfDNA test result.

In conclusion, this study shows that a detailed ultrasound examination performed after a cfDNA result that is high-risk for one of the common autosomal trisomies adds significantly to establishing an individualized risk. This is particularly true in cases with a high-risk result for trisomies 18 and 13.

## References

[1] Kagan KO, Sonek J, Kozlowski P (2022). Antenatal screening for chromosomal abnormalities. Arch Gynecol Obstet.

[2] Rose NC, Barrie ES, Malinowski J, Jenkins GP, McClain MR, LaGrave D (2022). Systematic evidence-based review: the application of noninvasive prenatal screening using cell-free DNA in general-risk pregnancies. Genet Med.

[3] Wagner P, Sonek J, Hoopmann M, Abele H, Kagan KO (2016). First-trimester screening for trisomies 18 and 13, triploidy and Turner syndrome by detailed early anomaly scan. Ultrasound Obstet Gynecol.

[4] Moran CJ, Tay JB, Morrison JJ (2002). Ultrasound detection and perinatal outcome of fetal trisomies 21, 18 and 13 in the absence of a routine fetal anomaly scan or biochemical screening. Ultrasound Obstet Gynecol.

[5] Cho RC, Chu P, Smith-Bindman R (2009). Second trimester prenatal ultrasound for the detection of pregnancies at increased risk of Trisomy 18 based on serum screening. Prenat Diagn.

[6] Agathokleous M, Chaveeva P, Poon LCY, Kosinski P, Nicolaides KH (2013). Meta-analysis of second-trimester markers for trisomy 21. Ultrasound Obstet Gynecol.

[7] Abele H, Wagner P, Sonek J, Hoopmann M, Brucker S, Artunc-Ulkumen B (2015). First trimester ultrasound screening for Down syndrome based on maternal age, fetal nuchal translucency and different combinations of the additional markers nasal bone, tricuspid and ductus venosus flow. Prenat Diagn.

[8] Prodan NC, Wiechers C, Geipel A, Walter A, Siegmann HJ, Kozlowski P (2022). Universal cell free DNA or contingent screening for Trisomy 21: does it make a difference? A comparative study with real data. Fetal Diagn Ther.

[9] Kagan KO, Hoopmann M, Pfaff T, Prodan N, Wagner P, Schmid M (2020). First trimester screening for common trisomies and microdeletion 22q11.2 syndrome using cell-free DNA: a prospective clinical study. Fetal Diagn Ther.

[10] Kagan KO, Sonek J, Sroka A, Abele H, Wagner P, Prodan N (2019). False-positive rates in screening for trisomies 18 and 13: a comparison between first-trimester combined screening and a cfDNA-based approach. Arch Gynecol Obstet.

[11] Kagan KO, Wagner P, Hoopmann M, Abele H (2019). First trimester screening based on ultrasound and cfDNA vs. first-trimester combined screening with additional ultrasound markers. Eur J Obstet Gynecol Reprod Biol.

[12] Kagan KO, Maier V, Sonek J, Abele H, Lüthgens K, Schmid M (2018). False-positive rate in first-trimester screening based on ultrasound and cell-free DNA versus first-trimester combined screening with additional ultrasound markers. Fetal Diagn Ther.

[13] Kagan KO, Sroka F, Sonek J, Abele H, Lüthgens K, Schmid M (2018). First-trimester risk assessment based on ultrasound and cell-free DNA vs combined screening: a randomized controlled trial. Ultrasound Obstet Gynecol.

[14] Bilardo CM, Chaoui R, Hyett JA, Kagan KO, Karim JN, Papageorghiou AT, Poon LC, Salomon LJ, Syngelaki A, Nicolaides KH (2023). ISUOG practice guidelines (updated): performance of 11–14-week ultrasound scan. Ultrasound Obstet Gynecol.

[15] Kozlowski P, Burkhardt T, Gembruch U, Gonser M, Kähler C, Kagan KO (2019). DEGUM, ÖGUM, SGUM and FMF Germany recommendations for the implementation of first-trimester screening, detailed ultrasound, cell-free DNA screening and diagnostic procedures. Ultraschall Der Medizin Eur J Ultrasound.

[16] von Kaisenberg C, Chaoui R, Häusler M, Kagan K, Kozlowski P, Merz E (2016). Quality requirements for the early fetal ultrasound assessment at 11–13+6 weeks of gestation (DEGUM levels II and III). Ultraschall in der Medizin Eur J Ultrasound.

[17] Merz E, Eichhorn KH, von Kaisenberg C, Schramm T, der Degum-Stufe III A (2012). Updated quality requirements regarding secondary differentiated ultrasound examination in prenatal diagnostics (= DEGUM level II) in the period from 18 + 0 to 21 + 6 weeks of gestation. Ultraschall in der Medizin (Stuttgart, Germany : 1980).

[18] Navaratnam K, Alfirevic Z, Gynaecologists the RC of O and (2022). Amniocentesis and chorionic villus sampling. BGOG Int J Obstet Gynaecol.

[19] Okoror CEM, Arora S (2023). Prenatal diagnosis after high chance non-invasive prenatal testing for trisomies 21, 18 and 13, chorionic villus sampling or amniocentesis?—experience at a district general hospital in the United Kingdom. Eur J Obstet Gynecol Reprod Biol X.

[20] Kagan KO, Rosenberg R (2023) Wegweiser auffälliger NIPT. Ultraschall Med (Stuttg, Ger : 1980) 10.1055/a-2150-8253 37553079

[21] Hui L, Ellis K, Mayen D, Pertile MD, Reimers R, Sun L (2023). Position statement from the International Society for Prenatal Diagnosis on the use of non-invasive prenatal testing for the detection of fetal chromosomal conditions in singleton pregnancies. Prenat Diagn.

[22] Scott F, Smet M-E, Elhindi J, Mogra R, Sunderland L, Ferreira A (2023). Late first-trimester ultrasound findings can alter management after high-risk NIPT result. Ultrasound Obstet Gynecol.

[23] Zhen L, Li YJ, Yang YD, Li DZ (2019). The role of ultrasound in women with a positive NIPT result for trisomy 18 and 13. Taiwan J Obstet Gynecol.

[24] Lüthgens K, Häbig K, Sonek J, Kagan KO (2023). Screen-positive rate in cell free DNA screening for trisomy 21. Prenat Diagn.

[25] Lüthgens K, Sinzel M, Kolar M, Kagan KO (2023). Screen-positive rate in cell-free DNA screening for microdeletion 22q11.2. Prenat Diagn.

[26] Wright D, Wright A, Nicolaides KH (2015). A unified approach to risk assessment for fetal aneuploidies. Ultrasound Obstet Gynecol.

[27] Kagan K, Tercanli S, Hoopmann M (2021). Ten reasons why we should not abandon a detailed first trimester anomaly scan. Ultraschall Der Medizin Eur J Ultrasound.

[28] Bardi F, Kagan KO, Bilardo CM (2023). First-trimester screening strategies: a balance between costs, efficiency and diagnostic yield. Prenat Diagn.

[29] Maya I, Sheelo LS, Brabbing-Goldstein D, Matar R, Kahana S, Agmon-Fishman I (2022). Residual risk for clinically significant copy number variants in low-risk pregnancies, following exclusion of noninvasive prenatal screening–detectable findings. Am J Obstet Gynecol.

[30] Galeva S, Gil MM, Konstantinidou L, Akolekar R, Nicolaides KH (2019). First-trimester screening for trisomies by cfDNA testing of maternal blood in singleton and twin pregnancies: factors affecting test failure. Ultrasound Obstet Gynecol.

